# The TALE Class Homeobox Gene *Smed-prep* Defines the Anterior Compartment for Head Regeneration

**DOI:** 10.1371/journal.pgen.1000915

**Published:** 2010-04-22

**Authors:** Daniel A. Felix, A. Aziz Aboobaker

**Affiliations:** Institute of Genetics, Queen's Medical Centre, University of Nottingham, United Kingdom; Harvard University, United States of America

## Abstract

Planaria continue to blossom as a model system for understanding all aspects of regeneration. They provide an opportunity to understand how the replacement of missing tissues from preexisting adult tissue is orchestrated at the molecular level. When amputated along any plane, planaria are capable of regenerating all missing tissue and rescaling all structures to the new size of the animal. Recently, rapid progress has been made in understanding the developmental pathways that control planarian regeneration. In particular Wnt/beta-catenin signaling is central in promoting posterior fates and inhibiting anterior identity. Currently the mechanisms that actively promote anterior identity remain unknown. Here, *Smed-prep*, encoding a TALE class homeodomain, is described as the first gene necessary for correct anterior fate and patterning during planarian regeneration. *Smed-prep* is expressed at high levels in the anterior portion of whole animals, and *Smed-prep(RNAi)* leads to loss of the whole brain during anterior regeneration, but not during lateral regeneration or homeostasis in intact worms. Expression of markers of different anterior fated cells are greatly reduced or lost in *Smed-prep(RNAi)* animals. We find that the ectopic anterior structures induced by abrogation of Wnt signaling also require *Smed-prep* to form. We use double knockdown experiments with the *S. mediterranea* ortholog of *nou-darake* (that when knocked down induces ectopic brain formation) to show that *Smed-prep* defines an anterior fated compartment within which stem cells are permitted to assume brain fate, but is not required directly for this differentiation process. *Smed-prep* is the first gene clearly implicated as being necessary for promoting anterior fate and the first homeobox gene implicated in establishing positional identity during regeneration. Together our results suggest that *Smed-prep* is required in stem cell progeny as they form the anterior regenerative blastema and is required for specifying anterior cell fates and correct patterning.

## Author Summary

Understanding the genetic basis of tissue regeneration (remaking) from adult structures is an important long-term goal for biomedical science. The widespread nature of regenerative phenomena in different animals allows us to study evolution's answers to coordinating this process. We use the relatively simple and experimentally tractable planarian model to study this process. After almost any amputation these animals unerringly replace all missing tissues. This ability has two key components. Firstly, planaria have a population of stem cells capable of rapidly dividing and becoming all the cell types that are missing, such as muscle, gut, and brain cells, after amputation. Secondly, the genetic information in these stem cells and the remaining tissue is able to coordinate the regeneration process so that new structures are the correct size and in the correct place. This allows the production of a fully functional individual at the end of the regeneration process. We are specifically interested in how structures end up in the correct place in new tissue they form. Here we discover and describe the role of a gene, called *Smed-prep*, particularly central to this process. *Smed-prep* is required to coordinate the regeneration of the planarian brain, arguably the most exciting part of planarian regeneration.

## Introduction

Planaria continue to blossom as a model system for understanding all aspects of regeneration [1]–[3]. A sustained and passionate effort by a number of scientists is pushing planaria to the forefront of the regeneration field, both technically [4], [5] and theoretically [6], and they are finally starting to be directly informative of phenomena in other systems [7]. They provide an opportunity to understand how the replacement of missing tissues from preexisting adult tissue is orchestrated at the molecular level. When amputated along any plane planaria are capable of regenerating all missing tissue and rescaling all structures to the new size of the animal [8].

Recent work has shown that conserved signaling pathways play a role in axial patterning during both regeneration and homeostatic tissue turnover [9]–[13]. In particular Wnt/beta-catenin signaling is necessary for posterior fate during regeneration, with loss of beta-catenin or Wnt signaling leading to all amputations regenerating anterior structures and a gradual loss of posterior identity during homeostasis [9], [10], [12]. Conversely, over activity of Wnt signaling induced by abrogating the expression of negative regulators of the pathways leads to ectopic posterior fate [9]. Further studies have begun to describe the temporal nature of this posterior specification circuit, as well the conserved nature of upstream regulation [14], [15].

Previously elegantly executed manipulative work has uncovered phenomena that suggest that anterior fated tissue can inhibit the regeneration of anterior fate elsewhere [3]. In addition some headway has been made in understanding the potential signaling systems responsible for this [16], [17]. In particular the planarian *nou-darake* (ndk) gene, an FGF-like receptor, has been shown to be necessary to restrict the formation of anterior-dorsal brain ganglia/cephalic ganglia (CG) to anterior regions [16]. Currently though nothing is known about the instructive signals required to promote anterior fate. We wished to uncover these signals that together must promote anterior fate and correctly pattern the brain as it reforms from stem cell progeny at anterior blastemas.

Given the involvement of conserved pathways already uncovered we hypothesized that other genetic circuits employed to specify positional domains in other animals would be responsible for this process during planarian regeneration. One obvious group of genes for this process would be planarian orthologs of the Hox genes and Hox gene co-factors, These are required for anterior-posterior axis specification in the metazoa [18], [19]. Planarian Hox orthologs have been previously studied, and in some cases are expressed in distinct spatial domains, but have as yet no functions are assigned to them in planaria.

This has led us to consider TALE class homeodomain containing genes, a subset of which act as Hox gene cofactors [18]. Collectively, they are known to modulate the activity of Hox proteins by regulating their localization within the cell and by increasing their binding site specificity, but also have many hox independent roles in development [20]–[23].

Here, *Smed-prep*, encoding a TALE class homeodomain, is described as the first gene that is necessary to instruct anterior fate and patterning during planarian regeneration.

## Results/Discussion

### *Smed-prep* encodes a TALE class homeodomain protein expressed in regeneration blastemas

**Figure 1:**
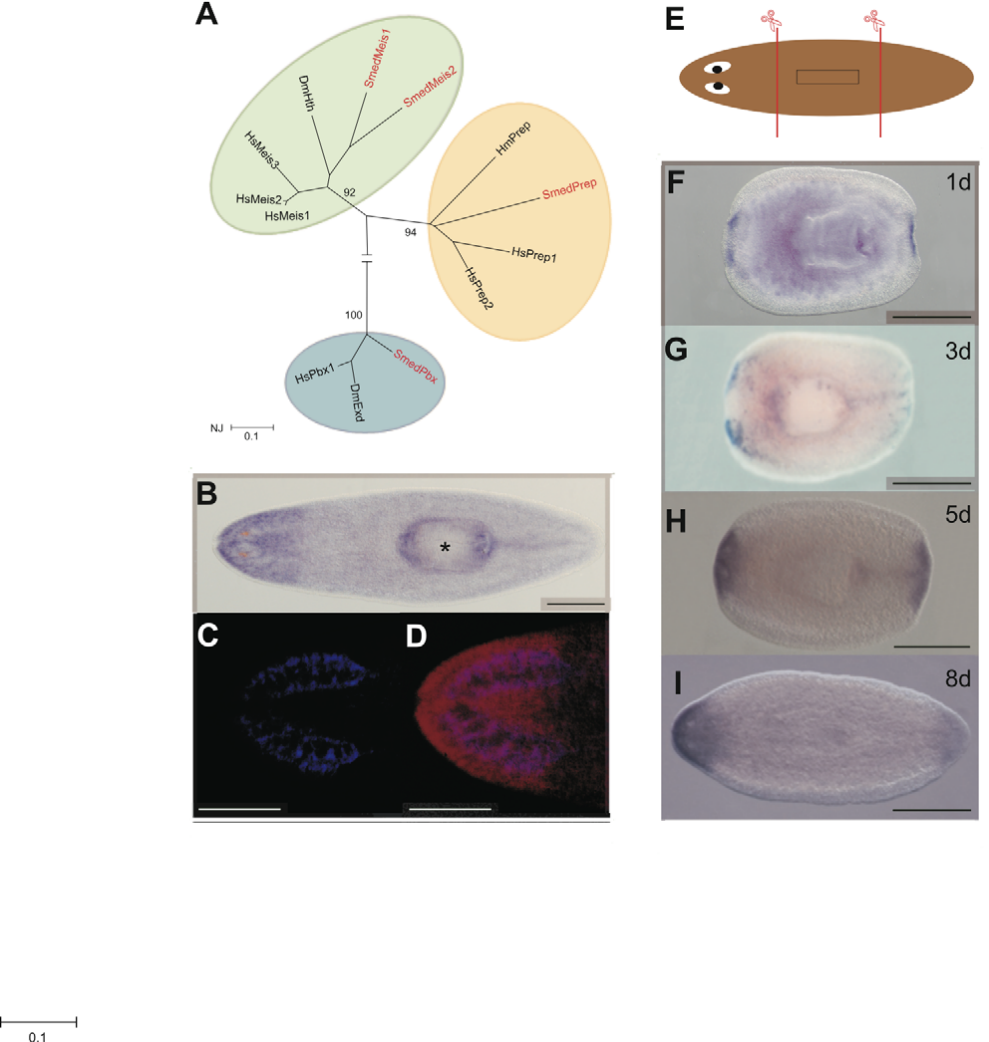
*Smed-Prep* encodes a TALE Homeobox gene expressed in regenerating blastemas. (A) Phylogenetic reconstruction of *S. mediterranea* TALE Class homeodomain proteins and representative orthologs, with most taxa removed for clarity (Hs: Homo sapien, Dm: *Drosophila melanogaster* Hm: *Hydra magnipapillata*), produced using a neighbor joining method and 500 bootstrap replicates. (B) *Smed-prep* expression in whole worms shows a distinct anterior domain of high expression. (C,D) demonstrate that the posterior margin of high *Smed-prep* expression coincides with posterior end of the brain. DAPI staining (blue) to highlight the brain (C) combined with false coloring of *Smed-prep* (red) expression (D). (E) Standard amputation protocol to assess expression during regeneration and regeneration phenotypes of RNAi experiments. Animals are cut pre- and post-pharyngeal to generate regenerating head, trunk and tail fragments. Expression of *Smed-prep* in regeneration blastemas is present in anterior and posterior blastemas in regenerating trunck pieces at 1 day (F), 3 days (G), 5 days (H), and 8 days (I) after amputation. Expression at 5 days clearly shows an absence of expression in the eye field, posterior expression at 8 days is reduced. All scale bars are 1 mm. Asterix indicates the pharynx.

The *Smed-prep* transcript was identified in an informatics screen for homeodomain proteins in the *Schmidtea mediterranea* genome. Searching the *S. mediterranea* genome identifies other TALE class homeodomain proteins [18], but *Smed-prep* encodes the only PREP ortholog (Figure 1A). The protein encoded by *Smed-prep* has high homology to other PREP proteins and contains the conserved features expected of this protein family (Figure S1). In vertebrates, PREP proteins have been implicated in a number of key developmental processes [23], including the correct patterning of anterior structures [21]. The function of Hox and Hox co-factors in planaria remains enigmatic. The fact that these two groups of homeodomains act together to pattern tissues in other systems makes them strong candidates for a role in providing positional information in planarians. For this reason we performed a detailed study of *Smed-prep*.

We performed *in situ* hybridization on whole and regenerating asexual planaria [24], [25]. We find that *Smed-prep* is expressed at ubiquitously low levels throughout the parenchyma and at higher levels in the head region. The posterior margin of anterior expression coincides with the most posterior position of cephalic ganglia (CG) (Figure 1C and 1D). We also detect low levels of *Smed-prep* expression in the posterior midline, at higher levels than the broad parenchymal expression, in approximately 50% (39/72) of animals (Figure 1B). *Smed-prep* expression is not sensitive to irradiation, indicating that *Smed-prep* is not expressed in, or dependent on, the ‘neoblast’ stem cells (data not shown). During regeneration induced by pre- and post-pharyngeal amputation (Figure 1E) *Smed-prep* expression is first detected at 24 h and is present in both anterior and posterior blastemas (Figure 1F). New *Smed-prep* expression is not detected at 6, 12 or 18 hours of regeneration. Expression in the anterior is bilateral up to 3 days but has expanded across the whole blastema at 5 days (Figure 1G and 1H). At 5 days *Smed-prep* is expressed throughout the anterior compartment with the notable exception of the eye field. We also detect feint expression in the posterior midline of approximately 50% of trunk fragments at 3 (18/41 fragments) and 5 days (23/40 fragments) of regeneration. We observe this in trunk fragments only (Figure 1G and 1H). This expression is absent later and presumably reappears after regeneration is complete and animals reach a homeostatic state (see above). At 8 days of regeneration, posterior blastema expression is reduced while expression in the anterior continues to be high (Figure 1I). This expression pattern led us to hypothesize a role for *Smed-prep* in patterning regenerating tissue after amputation. In particular expression in whole worms suggested that *Smed-prep* might have a role in pattering and/or maintaining anterior structures.

### *Smed-prep(RNAi)* results in loss of anterior structures specifically during anterior regeneration

**Figure 2:**
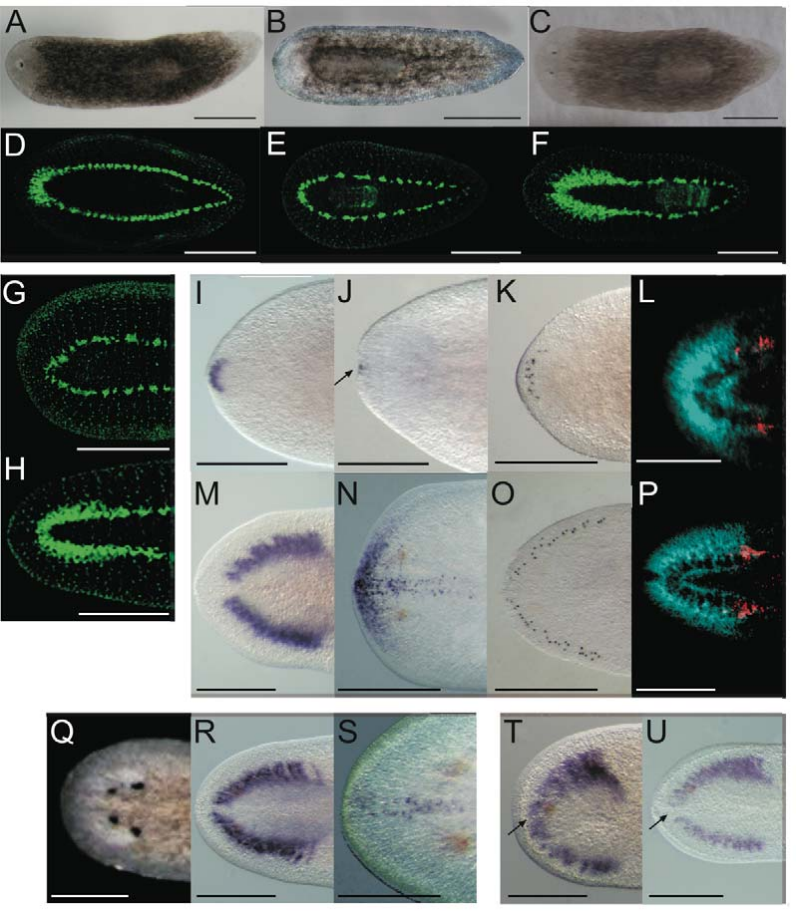
*Smed-prep(RNAi)* leads to the loss of anterior fate during regeneration. *Smed-prep(RNAi)* using a standard injecting and cutting protocol (Figure S2A) leads to animals with either one (A) or no eyes (B). Control *gfp(RNAi)* animals were all normal (C). Staining with the 3C11 monoclonal antibody to synapsin in *Smed-prep(RNAi)* with one eye (D), animals with no eyes (E), and *gfp(RNAi)* (F). *Smed-prep(RNAi)* animals (Figure S2B) (G) and *gfp(RNAi)* injected during regeneration. Staining with a probe to a glutamate receptor specific to CG/brain, branches, *Smed-GluR*, confirms reduction of CG structure to the most anterior tip (I). *Smed-sFRP-1*, a marker of anterior fate, is mostly absent or else confined to the very anterior tip (J). Staining with *cintillo* (K) shows that the number of these anterior cells is also reduced and restricted to the anterior tips of animals. Staining with the posterior brain marker *Smed-WntA* (red) shows that in animals where CG/brain is present A/P polarity of the brain (DAPI stained in blue) is maintained (L,P). *gfp(RNAi)* were normal for all these stains (M–P). Prolonged *Smed-prep(RNAi)* during homeostasis (Figure S2C) leads to the formation of two new eyes anterior to the original pair (Q) but not to any visible reduction or incorrect patterning of the CG/brain, as shown by *Smed-GluR* expression (R). The most anterior margin expression of *Smed-sFRP-1* is lost in *Smed-prep(RNAi)* homeostasis worms (S). *Smed-prep(RNAi)* worms amputated laterally (Figure S2A) are able to regenerate CG, as shown by *Smed-GluR* expression (T), but the regeneration is not patterned correctly as branches are fused (see arrow in T) compared to *gfp(RNAi)* animals (U). All panels depict 12 day regenerating trunks except: (G,H) 12 day regenerating tails, (Q,R,S) 28 days homeostasis after first injection, (T,U) 15 days regeneration after lateral regeneration. All scale bars 1 mm.

We performed RNAi [26], [27] of *Smed-prep* to investigate its function during regeneration (see Figure S2 for summary of injection protocols). *Smed-prep* dsRNA injection before inducing regeneration by amputation (Figure 1E) resulted in all worms having either a cyclops phenotype (Figure 2A) or no eyes at all (Figure 2B, Table 1). All animals had correct early blastema formation, normal levels of neoblast proliferation (data not shown) and no defects in posterior blastema formation (Figure 2A, 2B, 2D, and 2E). A similar cyclops phenotype has been described for a *S. mediterranea slit* ortholog [28]. Staining with an anti-arrestin VC-1 antibody specific for planarian photoreceptors and associated neurons [29] we observed that the single eye phenotype appeared to represent a fusion of two eyes (Figure S2D, S2E). We detected no other midline defects in regenerating animals that were described for *Smed-slit*, and *Smed-slit* expression itself was normal (Figure S2F and S2G). This suggests, in agreement with the *Smed-prep* expression pattern, that the cyclops phenotype is due to a defect in anterior patterning and fate rather than any midline defects. Control *gfp(RNAi)* animals had normal eye structure (Figure S2E).

**Table 1:** Summary of phenotypes for *Smed-prep(RNAi)* experiments.

Experiment	Nr. Exp.	Eye phenotypes	Smed-sFRP-1 Expression	Brain/CG presence (3C11, *Smed-GluR*)
*Smed-prep(RNAi)* trunks, 12dR	11	0%, 2 eyes 92%, 1 eye (389/424) 18%, no eye (35/424)	32% (8/25)*1	3c11 84% (27/32)*1 GluR 89% (33/37)*1
*Smed-prep(RNAi)* tails 12dR	11	0%, 2 eyes 58%, 1 eye (241/417) 42%, no eye (176/417)	0% (22/22)	3c11 47% (15/32)*1 GluR 51% (18/35)*1
*Smed-prep(RNAi)* in regenerating tails, 12dR	2	0%, 2 eyes 19%, 1 eye (4/26) 81%, no eye (21/26)	0% (9/9)	
*Smed-prep(RNAi)*, lateral regeneration, 15dR	2	36%, 2 eyes (9/25) 56%, 1 eye (14/25) 8%, no eye (2/25)		3c11 100% (9/9) GluR 100% (11/11)
*gfp(RNAi)* summary, 12dR, 15dR	15	100%, 2 eyes (350/351) 0%, 1 eye 0%, no eye (1/351)	100% (32/32)	3c11 100% (35/35) GluR 100% (42/42)
*Smed-prep(RNAi)* intact animals, 28d+ homeostasis	3	86%, 4 eyes (24/28) 14%, 2 eyes (4/28)	100% (6/6)*2	3c11 100% (8/8) GluR 100% (9/9)
*Smed-prep/gfp(RNAi)*, 12dR	3		17% (1/6)*1	GluR 75% (9/12)
*Smed-ndk/gfp(RNAi)*, 12dR	3	Ectopic eyes present	100% (16/16)	GluR 100% (14/14)*3
*Smed-ndk/prep(RNAi)*, 12dR	3	Ectopic eyes present	10% (2/20)*1	GluR 100% (15/15)*3
*Smed-gfp(RNAi)*, 12dR	3		100% (15/15)	GluR 100% (15/15)

*1 Strongly reduced expression.

*2 All retain some very weak expression in the longitudinal double row of cells; the normally far stronger expression along the anterior head margin is completely absent.

*3 Posteriorly expanded expression.

We investigated the structure of the planarian ventral nerve cords (VNCs) and CG using the anti-SYNORF1 (3C11) cross-reactive monoclonal antibody [30]. We found that in all *Smed-prep(RNAi)* animals the CG were greatly reduced, with almost no brain at all discernible in the most severely affected RNAi worms (Figure 2D and 2E). In these animals anti-SYNORF1 positive cells do form from differentiating neoblast progeny in the anterior as part of the VNCs. Significantly, anti-SYNORF1 positive cells are present along the whole anteroposterior axis. This suggests, along with correct pharynx and posterior regeneration that Smed-prep(RNAi) does not affect the general ability of stem cells to differentiate. All control *gfp(RNAi)* animals were normal (Figure 2C and 2F). We confirmed the loss of CG by looking at the expression of *Smed-GluR* (specific for CG (Figure 2I and 2M). This loss of anterior structures suggests a role for *Smed-prep* in patterning anterior structures and/or a requirement for *Smed-prep* in allowing neoblasts to differentiate into CG cells. This phenotype is different from that previously described for the *S. mediterranea* ortholog of *adenomatous polypolis coli* (APC), *a* negative regulator of Wnt signaling. *Smed-APC-1(RNAi)* results in ectopic posterior fate at anterior blastemas [9].

To build a more exact picture of the requirements for *Smed-prep* we also investigated its role during regeneration more directly. We injected regenerating animals after amputation and then re-amputated (Figure S2). This approach has previously been used as a proxy to separate regeneration specific effects from homeostatic effects [15]. Control *gfp(RNAi)* worms regenerated normally but *Smed-prep(RNAi)* worms failed to make eyes and CG almost entirely (Figure 2G and 2H, Table 1). All animals did regenerate normal VNCs within regenerated anterior tissue. This confirms that new *Smed-prep* expression during regeneration is required to properly replace anterior structures.

To investigate whether *Smed-prep* was required specifically for stem cell progeny to differentiate to CG or instead primarily for global anterior fates we investigated the expression of *cintillo* [31] and *Smed-sFRP-1* [9], [12]. These genes represent two different anterior markers that are not expressed in CG cells. We find that both *cintillo* and *Smed-sFRP-1* expression are greatly reduced or absent in *Smed-prep(RNAi)* animals at 12 days of regeneration (Figure 2J and 2K). In the case of *Smed-sFRP-1* expression we observed a correlation between the strength of the *Smed-prep(RNAi)* phenotype and whether any *Smed-sFRP-1* expression was detectable. Those animals that maintained a single eye (and therefore some CG) also had some remaining *Smed-sFRP-1* expression. Animals with stronger phenotypes (no eyes) had no detectable anterior *Smed-sFRP-1* expression. All *gfp(RNAi)* animals had normal expression for both these markers (Figure 2N and 2O). Together these data suggest that *Smed-prep* is required for correct anterior blastema fate patterning during regeneration, rather than solely for CG formation by differentiating neoblasts.

This loss of anterior markers led us to consider whether *Smed-prep(RNAi)* leads to a homeotic like posteriorisation of the planarian body plan. We found no evidence for this by looking at the relative position of the regenerating or fully formed pharynx, the expression of a medial marker *Smed-Tcen49* [32], or by looking at the expression of posterior markers such as *Smed-HoxD* [10]. Thus we infer that *Smed-prep(RNAi)* leads to a reduction in the formation of anterior structures, but neither a change to posterior fate at anterior blastemas nor an expansion in posterior or medial fates in existing tissues (Figure S2J, S2K, S2L, and S2M). We also found that early Smed-*sFRP-1* expression at anterior blastemas at 24 hours of regeneration is absent in *Smed-prep(RNAi)* animals. This suggests *Smed-prep* acts to provide anterior fate and pattern the anterior blastema, after polarity is set (Figure S2H and S2I).

The planarian brain and the planarian head have distinct A/P polarity, as is the case in other animals [17]. *Smed-prep* expression is higher in the anterior and lateral margins of the planarian head (Figure 1B). We wished to know whether this was a reflection of *Smed-prep* having a role in defining different A/P fates within the anterior blastema itself. In this case any remaining brain fated tissues observed in *Smed-prep(RNAi)* animals (Table 1) would be expected to have posterior brain fate. By investigating the expression of *Smed-WntA*, a marker of the posterior brain [17] we found that *Smed-prep(RNAi)* animals that regenerated one eye and some CG also maintained antero-posterior identity within their much reduced anterior structures (Figure 2L and 2P). In these animals Smed-*WntA* still labels a posterior domain of the remaining CG. This suggests that *Smed-prep* is required to specify an anterior field of cells in which further A/P patterning occurs.

### *Smed-prep* is required for anterior patterning but not for brain maintenance or regeneration during homeostasis or lateral regeneration

We performed long term *Smed-prep(RNAi)* in whole worms, to assess its role during normal homeostasis and tissue turnover. Long-term knockdown did not result in loss or proportional reduction of anterior structures or CG/Brain (Figure 2R, Table 1). However, *Smed-prep(RNAi)* worms developed a new pair of photoreceptors anterior to the original pair (Figure 2Q). This result suggests that *Smed-prep* expression in the anterior of whole worms is required for correct positioning of the photoreceptors during homeostasis but not for CG maintenance. *Smed-sFRP-1* expression was also affected in these animals, with loss of anterior margin and lateral expression, but maintenance of weaker ventral antero-medial expression (Figure 2S). This provides more evidence to suggest that *Smed-sFRP-1* expression is dependent on *Smed-prep* expression. These data show that *Smed-prep* has different roles in establishing anterior structures and their subsequent maintenance.

The finding that the CG were not reduced in homeostasis led us to consider whether *Smed-prep(RNAi)* would affect the lateral regeneration of anterior structures. We reasoned that if *Smed-prep* was not required for CG maintenance during homeostasis, then alternative anterior maintenance mechanisms must be active during homeostasis. These alternate mechanisms could also be sufficient to orchestrate lateral regeneration, a scenario where existing anterior structures are left partially intact. We cut *Smed-prep(RNAi)* worms longitudinally (Figure S2A) and observed regeneration. We found that *Smed-prep(RNAi)* worms were able to laterally regenerate all structures, with correct scaling, and subsequent normal behavior. While some worms did not regenerate a second eye correctly, all animals regenerated lateral CG. However, on looking at the pattern of the CG structure in more detail we noticed that the bilateral CG fused at the anterior tip (Figure 2T and 2U). In this regenerative scenario *Smed-prep(RNAi)* animals can regenerate antero-laterally but CG structures are not patterned correctly. This indicates that while *Smed-prep* is specifically required for the replacement of missing anterior structures when they are absent, it is not required to generate missing anterior fated structures during antero-lateral regeneration, i.e. when one side of the brain is still present. Instead, it is only required for the formation of correct pattern during this regenerative scenario. It seems likely that the remaining anterior tissue contains cues, generated downstream of *Smed-prep* during normal anterior regeneration, that are sufficient to direct neoblast progeny to CG fate.

### Double *Smed-prep*/*nou-darake(RNAi)* shows that *Smed-prep* is required for anterior patterning but not for brain differentiation

**Figure 3:**
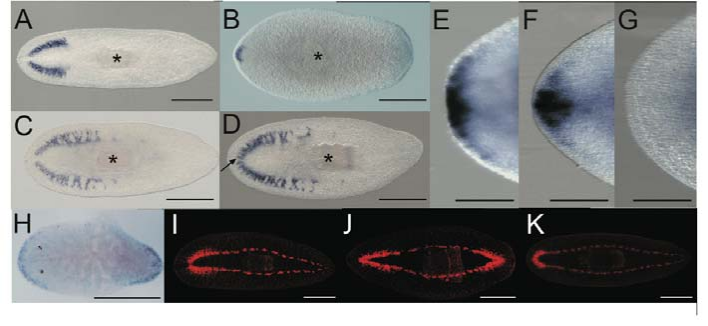
Double *Smed-prep/Smed-ndk(RNAi)* and double *Smed-prep/Smed-beta-catenin-1(RNAi)* phenotypes further define the role of *Smed-prep.* *Smed-GluR* expression in *gfp(RNAi)* (A), *Smed-prep/gfp(RNAi)* (B), *Smed-ndk/gfp(RNAi)* (C), and *Smed-prep/ndk(RNAi)* (D) animals. *Smed-prep/ndk(RNAi)* (D) animals have ectopic CG cells and have fused bilateral CG branches (arrow). *Smed-prep/ndk(RNAi)* (G) animals also fail to correctly express the anterior marker *Smed-sFRP-1*, which is expressed in *gfp(RNAi)* (E) and *Smed-ndk/gfp(RNAi)* (F) animals. *Smed-beta-catenin-1(RNAi)* animals (H) ectopically express *Smed-prep* at the “new” anterior end and *Smed-beta-catenin-1/gfp(RNAi)* animals regenerate heads at both blastemas of regenerating fragments (J). The regeneration of anterior structures is greatly reduced or entirely absent in posterior blastemas in *Smed- prep/beta-catenin-1(RNAi)* (K) and regneration is normal control (I) *gfp(RNAi)* animals, whereas the regenerated head in *Smed-prep/beta-catenin-1(RNAi)* shows the expected head reduction of *Smed- prep(RNAi)*. All panels are trunk pieces accept (H) which is a head. All pieces are 15 day regenerants. All scales bars are 1 mm except (E–G) which are 500 µm.

Our experiments thus far suggest that *Smed-prep* is required for anterior patterning and fate. To formally rule out the possibility that *Smed-prep* is also directly required during anterior regeneration for stem cell differentiation into CG we utilized the previously described *nou-darake* (*ndk*) RNAi phenotype [16]. RNAi of this FGF-like receptor gene leads to ectopic posterior expansion of CG during homeostasis and regeneration. We predicted that if *Smed-prep* was required for anterior patterning but not for neoblast differentiation then double *Smed-prep/ndk(RNAi)* worms would display expanded CG differentiation, but with aberrant anterior patterning and loss of anterior marker expression. *Smed-prep/gfp(RNAi)* and *Smed-ndk/gfp(RNAi)* animals regenerated with reduced and expanded CG respectively compared to *gfp(RNAi)* worms (Figure 3B and 3C). *Smed-prep/ndk(RNAi)* animals had expanded CG but this expansion was patterned incorrectly (Figure 3D). The CG of *Smed-prep/Smed-ndk(RNAi)* animals are fused at the anterior tip, similar to *Smed-prep(RNAi)* laterally regenerated animals (Figure 3D). Both *gfp(RNAi)* and *smed-ndk/gfp(RNAi)* animals have normally patterned bilateral CG (Figure 3A and 3C). To test if this mispatterning was concomitant with the loss of anterior fate we also looked at *Smed-sFRP-1* expression. Whereas *Smed-sFRP-1* expression was normal in *Smed-ndk(RNAi)* animals after regeneration it was absent or greatly reduced in *Smed-prep/ndk(RNAi)* animals (Figure 3E–3G). This suggests that *Smed-prep* specifies an anterior domain during regeneration and that stem cell progeny normally differentiate to form CG only within this domain. This restriction requires activity of *Smed-ndk*, which is also expressed in an anterior domain. In double *Smed-prep/ndk(RNAi)* animals the loss of *Smed-ndk* removes this restriction on neoblast progeny, allowing them to adopt CG fate without the presence of *Smed-prep* expression, but does not rescue the defects in anterior patterning.

### *Smed-prep* is required for formation of ectopic anterior structures in *Smed-beta-catenin-1(RNAi)* animals

Wnt signaling is central in patterning the antero-posterior axis of planarians by promoting posterior fate [9], [10], [12], [15]. Given the finding that *Smed-prep* is not required for CG maintenance or formation during homeostasis and lateral regeneration respectively, it remained unclear whether *Smed-prep* would be required for the ectopic anterior structures observed when Wnt signaling is attenuated. We found that when *Smed-beta-catenin-1(RNAi)* results in head regeneration at both anterior and posterior blastemas [3]–[5], ectopic and prolonged expression of *Smed-prep* in these new heads is observed (Figure 3H). In addition *Smed-prep/beta-catenin-1*(RNAi) reduced anterior structures at both ends (Figure 3K). As *Smed-prep* expression is initially present at both posterior and anterior blastemas our data suggest that active Wnt signaling in the posterior blastema suppresses *Smed-prep* action at posterior blastemas post-transcriptionally.

*Smed-prep* is the first gene clearly implicated as being necessary for promoting anterior fate during regeneration in *S. mediterranea*. We propose that after initial polarity determination, involving Wnt signals and other as yet unknown mechanisms, *Smed-prep* expression in neoblast progeny determines an anterior field of cells in which anterior structures differentiate and are patterned. At posterior blastemas *Smed-prep* activity is inhibited post-transcriptionally by Wnt activity. This now provides the opportunity to discover downstream genes that are required for further fine patterning during anterior regeneration, as some of these are likely transcriptional targets of *Smed-prep* activity.

In other animals the function of PREP TALE class homeodomains remains rather poorly defined compared to those of other TALE class family genes. In the both major invertebrate genetics models, *C. elegans* and *D. melanogaster*, a direct ortholog of PREP TALE class homeodomains is absent [18]. Interestingly both worms and flies contain MEIS orthologs (*unc-62* and *homothorax* respectively) that have broad roles in specifying fate during development [33], [34] and other members of the nematode and arthropod phyla do have PREP orthologs [18]. The finding that PREP is involved in zebra fish brain development may suggest that PREP has an evolutionary conserved role in anterior fates. Broader phylogenetic study of its function is required to test this [21]. Here, we show that *Smed-prep* expression and function delineates the whole anterior domain, including all regions of the brain. Previous studies of Hox and Hox co-factor function have not implicated these two groups of genes in defining the most anterior structures of other vertebrates [35] or arthropods [36].

Significantly, the requirement for *Smed-prep* is observably different during homeostasis and different regenerative scenarios. This illustrates that the genetic networks available to solve different regenerative scenarios may be diverse and are likely to depend on the informational/signaling capacity of the differentiated portion of starting tissue. In addition it is the first time that homeobox transcription factors have been directly implicated in A/P patterning in planaria. We suspect that other conserved homeodomain proteins will also play core roles in specifying positional information during regeneration.

## Materials and Methods

### Animals

All experiments were performed with a clonal line originally generated from a single animal of the asexual strain of the planarian *S. mediterranea* collected in Montjuïc (provided by Professor Emili Saló i Boix) maintained at 20°C in tap water treated with activated charcoal and buffered with 0.5 ml/L 1 M NaHCO3. Planarians were fed veal liver and starved for at least one week prior to experiments.

### Isolation of *Smed-prep*

To identify planarian homologues of TALE transcription factors we searched a local database of Version 3.1 of the *S. mediterranea* Genome Project for orthologs of mammalian TALE genes (http://genome.wustl.edu/genomes). The contigs 018898 and 020093 containing *Smed-prep* were analyzed using Vector NTI (Invitrogen) and sequence data supplemented by using RACE (Ambion RLM Race Kit). The primers Sm-Prep-Forward with sequence ATTGCTACTAGAGCAATGTGAACAAGC and Sm-Prep-Reverse with sequence ATTCTGCGTCGGGCATTGAT amplify a 810 bp fragment which was used for whole mount ISH hybridization and RNAi knockdown. PREP and TALE proteins sequences were taken from *Mukherjee at al* [18] and alignments checked with the CLUSTAL [37]. Phylogenetic reconstruction was conducted using *MEGA* version 4 using the bootstrapped neighbour-joining method [38]. The *Smed-prep* sequence has been submitted to GenBank with accession number GU290186.

### RNAi

DsRNAs were synthesized as described previously [39]. Control animals were injected with dsRNA of GFP that has no homology in the planarian genome. DsRNA microinjection was performed as described elsewhere [27]. For injection schedules please refer to Figure S2. For double RNAi experiments concentrations for each gene were maintained at 1 µg/µl after mixing and for GFP controls 2 µg/µl was injected.

### Whole-mount ISH hybridization, immuno-staining, and imaging

Whole mount ISH hybridization was carried out as described previously [25] with modifications described in [40] and [24]. The paraformaldehyde solution for the fixation step was prepared fresh and adjusted to pH 9.5 using NaOH.

For immuno-staining animals were killed in 2% HCl for 5 min on ice and then fixed in Carnoy's solution for 2 h at 4°C. After fixation, samples were processed as described elsewhere [41], [42]. The following primary antibodies were used: anti-SYNORF1, a mouse monoclonal antibody specific for synapsin (Developmental Studies HybridomaBank, dilution of 1∶25) and anti-arrestin VC-1, a mouse monoclonal antibody specific for planarian photosensitive cells (kindly provided by Hidefumi Orii, used at a dilution of 1∶15,000). Goat anti-mouse secondary antibody conjugated to Alexa 488 or Alexa 546 (Molecular Probes) was used at a 1∶400 dilution.

Brightfield pictures were taken on a Zeiss Discovery V8 from CarlZeiss using an AxioCam MRC from CarlZeiss. Fluorescent pictures were taken on a Leica MZ16F fluorescence stereomicroscope using a Leica DFC 300Fx camera (Leica Lasertechnik, Heidelberg). Confocal laser scanning microscopy was performed with a LeicaSP2 confocal laser scanning microscope (CLSM) (Leica Lasertechnik, Heidelberg).

## Supporting Information

Figure S1 Alignment of *Smed-prep* translation to other animal PREP proteins. Alignment of *Smed-Prep* across the conserved MEIS and Homeodomain regions of this TALE class protein with other animals. The *Smed-Prep* translation is underlined in red.
(0.03 MB PDF)

Figure S2 RNAi protocols and characterization of *Smed-prep* function. Explanation of RNAi injection schemes and further analysis of *Smed-prep* function. Figurative explanation of RNAi injection and amputation protocols used for assaying *Smed-prep* function. In the standard protocol animals receive 3×32 nl injections of dsRNA at 1 μg/μl for three consecutive days before pre- and post- pharyngeal or longitudinal amputations are performed (A). To assay the effect of *Smed-prep(RNAi)* specifically during regeneration animals tails are amputated and injected 3 times with 3×32 nl injections of dsRNA at 1 μg/μl as depicted. The animals are then re-amputated (B). Homeostasis experiments were conducted for 28 days or longer. Initially animals were injected as in (A) but instead of being amputated they were left intact, fed and injected with a single set of 3×32 nl injections of dsRNA at 1 μg/μl for the subsequent weeks. Staining with the anti-arrestin VC-1 monoclonal antibody against the photoreceptor neurons shows that *Smed-prep(RNAi)* animals have only one photoreceptor, which appears to be a fusion of two normal eyes (D). *gfp(RNAi)* animals always regenerate a normal visual system (E). The midline of *Smed-prep(RNAi)* animals (G) seems normal and *Smed-slit* expression that labels cells in the midline of *gfp(RNAi)* animals (F) is unaffected. The expression of Smed-sFRP-1 appears early during anterior regeneration. At 24 hours of regeneration it can already be seen in the blastema in *gfp(RNAi)* animals (H). In *Smed-prep(RNAi)* animals expression is not detected in tail pieces even when the sample is left to develop until background is very high (I). The expression of *HoxD* is detected in the tail parenchyma up to the mouth of the pharynx, in the mouth itself and in a few scattered cells just anterior to the pharynx in *gfp(RNAi)* animals (J). There is no ectopic expression detected in the head of *Smed-prep(RNAi)* animals (K). The normal expression domain of *Smed-Tcen49* in scattered cell clusters in the trunk region of the planaria (L) is not expanded anteriorly in *Smed-prep(RNAi)* animals (M).
(0.52 MB PDF)

## References

[1] Agata K, Umesono Y (2008). Brain regeneration from pluripotent stem cells in planarian.. Philos Trans R Soc Lond B Biol Sci.

[2] Reddien PW, Sanchez Alvarado A (2004). Fundamentals of planarian regeneration.. Annu Rev Cell Dev Biol.

[3] Salo E (2006). The power of regeneration and the stem-cell kingdom: freshwater planarians (Platyhelminthes).. Bioessays.

[4] Reddien PW, Bermange AL, Murfitt KJ, Jennings JR, Sanchez Alvarado A (2005). Identification of genes needed for regeneration, stem cell function, and tissue homeostasis by systematic gene perturbation in planaria.. Dev Cell.

[5] Reddien PW, Oviedo NJ, Jennings JR, Jenkin JC, Sanchez Alvarado A (2005). SMEDWI-2 is a PIWI-like protein that regulates planarian stem cells.. Science.

[6] Umesono Y, Agata K (2009). Evolution and regeneration of the planarian central nervous system.. Dev Growth Differ.

[7] Glazer A, Wilkinson A, Backer CB, Lapan S, Gutzman JH (2009). The Zn Finger protein Iguana impacts Hedgehog signaling by promoting ciliogenesis.. Dev Biol.

[8] Morgan TH (1900). Regeneration in Planarians.. Archiv Fur Entwicklungsmechanik Der Organismen (copy in filing cabinet).

[9] Gurley KA, Rink JC, Sanchez Alvarado A (2008). Beta-catenin defines head versus tail identity during planarian regeneration and homeostasis.. Science.

[10] Iglesias M, Gomez-Skarmeta JL, Salo E, Adell T (2008). Silencing of Smed-{beta}catenin1 generates radial-like hypercephalized planarians.. Development.

[11] Molina MD, Salo E, Cebria F (2007). The BMP pathway is essential for re-specification and maintenance of the dorsoventral axis in regenerating and intact planarians.. Dev Biol.

[12] Petersen CP, Reddien PW (2008). Smed-betacatenin-1 is required for anteroposterior blastema polarity in planarian regeneration.. Science.

[13] Reddien PW, Bermange AL, Kicza AM, Sanchez Alvarado A (2007). BMP signaling regulates the dorsal planarian midline and is needed for asymmetric regeneration.. Development.

[14] Adell T, Salo E, Boutros M, Bartscherer K (2009). Smed-Evi/Wntless is required for beta-catenin-dependent and -independent processes during planarian regeneration.. Development.

[15] Petersen CP, Reddien PW (2009). A wound-induced Wnt expression program controls planarian regeneration polarity.. Proc Natl Acad Sci U S A.

[16] Cebria F, Kobayashi C, Umesono Y, Nakazawa M, Mineta K (2002). FGFR-related gene nou-darake restricts brain tissues to the head region of planarians.. Nature.

[17] Kobayashi C, Saito Y, Ogawa K, Agata K (2007). Wnt signaling is required for antero-posterior patterning of the planarian brain.. Dev Biol.

[18] Mukherjee K, Burglin TR (2007). Comprehensive analysis of animal TALE homeobox genes: new conserved motifs and cases of accelerated evolution.. J Mol Evol.

[19] Ryan JF, Mazza ME, Pang K, Matus DQ, Baxevanis AD (2007). Pre-bilaterian origins of the Hox cluster and the Hox code: evidence from the sea anemone, Nematostella vectensis.. PLoS ONE.

[20] Berthelsen J, Zappavigna V, Ferretti E, Mavilio F, Blasi F (1998). The novel homeoprotein Prep1 modulates Pbx-Hox protein cooperativity.. EMBO J.

[21] Deflorian G, Tiso N, Ferretti E, Meyer D, Blasi F (2004). Prep1.1 has essential genetic functions in hindbrain development and cranial neural crest cell differentiation.. Development.

[22] Laurent A, Bihan R, Omilli F, Deschamps S, Pellerin I (2008). PBX proteins: much more than Hox cofactors.. Int J Dev Biol.

[23] Moens CB, Selleri L (2006). Hox cofactors in vertebrate development.. Dev Biol.

[24] Gonzalez-Estevez C, Arseni V, Thambyrajah RS, Felix DA, Aboobaker AA (2009). Diverse miRNA spatial expression patterns suggest important roles in homeostasis and regeneration in planarians.. Int J Dev Biol.

[25] Umesono Y, Watanabe K, Agata K (1997). A planarian orthopedia homolog is specifically expressed in the branch region of both the mature and regenerating brain.. Dev Growth Differ.

[26] Fire A, Xu S, Montgomery MK, Kostas SA, Driver SE (1998). Potent and specific genetic interference by double-stranded RNA in Caenorhabditis elegans.. Nature.

[27] Sanchez Alvarado A, Newmark PA (1999). Double-stranded RNA specifically disrupts gene expression during planarian regeneration.. Proc Natl Acad Sci U S A.

[28] Cebria F, Guo T, Jopek J, Newmark PA (2007). Regeneration and maintenance of the planarian midline is regulated by a slit orthologue.. Dev Biol.

[29] Sakai F, Agata K, Orii H, Watanabe K (2000). Organization and regeneration ability of spontaneous supernumerary eyes in planarians -eye regeneration field and pathway selection by optic nerves.. Zoolog Sci.

[30] Cebria F (2008). Organization of the nervous system in the model planarian Schmidtea mediterranea: an immunocytochemical study.. Neurosci Res.

[31] Oviedo NJ, Newmark PA, Sanchez Alvarado A (2003). Allometric scaling and proportion regulation in the freshwater planarian Schmidtea mediterranea.. Dev Dyn.

[32] Bueno D, Vispo M, Sancho V, Romero R (2001). Maintenance of A/P body regions in planarians by tcen49, a putativ cystine-knot neurotrophin.. Belg J Zool.

[33] Van Auken K, Weaver D, Robertson B, Sundaram M, Saldi T (2002). Roles of the Homothorax/Meis/Prep homolog UNC-62 and the Exd/Pbx homologs CEH-20 and CEH-40 in C. elegans embryogenesis.. Development.

[34] Aldaz S, Morata G, Azpiazu N (2005). Patterning function of homothorax/extradenticle in the thorax of Drosophila.. Development.

[35] Wilson SW, Houart C (2004). Early steps in the development of the forebrain.. Dev Cell.

[36] Lynch JA, Brent AE, Leaf DS, Pultz MA, Desplan C (2006). Localized maternal orthodenticle patterns anterior and posterior in the long germ wasp Nasonia.. Nature.

[37] Higgins DG, Thompson JD, Gibson TJ (1996). Using CLUSTAL for multiple sequence alignments.. Methods Enzymol.

[38] Tamura K, Dudley J, Nei M, Kumar S (2007). MEGA4: Molecular Evolutionary Genetics Analysis (MEGA) software version 4.0.. Mol Biol Evol.

[39] Boutros M, Kiger AA, Armknecht S, Kerr K, Hild M (2004). Genome-wide RNAi analysis of growth and viability in Drosophila cells.. Science.

[40] Nogi T, Levin M (2005). Characterization of innexin gene expression and functional roles of gap-junctional communication in planarian regeneration.. Dev Biol.

[41] Basyuk E, Bertrand E, Journot L (2000). Alkaline fixation drastically improves the signal of in situ hybridization.. Nucleic Acids Res.

[42] Cebria F, Newmark PA (2005). Planarian homologs of netrin and netrin receptor are required for proper regeneration of the central nervous system and the maintenance of nervous system architecture.. Development.

