# Supplementary material for: The TALE Class Homeobox Gene Smed-prep Defines the Anterior Compartment for Head Regeneration
Source: PLoS Genet. 2010 Apr 22;6(4):e1000915. doi: 10.1371/journal.pgen.1000915 (PMC2858555; doi:10.1371/journal.pgen.1000915)
Supplement: Figure S1 — Alignment of Smed-prep translation to other animal PREP proteins. Alignment of Smed-Prep across the conserved MEIS and Homeodomain regions of this TALE class protein with other animals. The Smed-Prep translation is underlined in red. (0.03 MB PDF) [file pgen.1000915.s001.pdf]

Figure S1, related to Figure 1

A

Sequence alignment of the MEIS domain

```
Prep2 H.sapiens     1 QAQLEADKRAVYRHPLFPLLTLLFEKCEQATQGSECITSASFDVDIENFVHQQEQEHKPF
Prep2 M.musculus    1 QAQLEADKRAVYRHPLFPLLTLLFEKCEQATQGSECITSASFDVDIENFVHQQEQEHKPF
Prep1 H.sapiens     1 QTPMDVDKQAIYRHPLFPLLALLFEKCEQSTQGSEGTTSASFDVDIENFVRKQEKEGKPF
Prep1 M.musculus    1 QTPMDADKQAIYRHPLFPLLALLFEKCEQSTQGSEGTTSASFDVDIENFVRKQEKDGKPF
X.tropicalis        1 QAQLEADKRAVYRHPLFPLLTLLFEKCEQATQGSECITSASFDVDIENFVHQQEQEHKPF
Prep1.1 D.rerio     1 QTQMDIDKACIYRHPLFPLLALLFEKCEQSTLGSDCVTSASFDVDIENFVRSQEKDGKAF
Smed-prep           1 ADGLEKEKKSIYGHPLYPILSLLLEQCEQATASPDSQSPDTFEADLQSYILRNDNNEKYF
C.intestinalis      1 AAQLENDKHLIKTHPLFVLLELLFEKCERATRGEDNPTSLSFDDDIQEFVRREDFRMSPI
T.castaneum         1 QAQFEADKRAVYKHPLFPLLALLFERCELATQSSDPQSSDAFNLDIQAFVQHQERDRKPF
A.mellifera         1 QAQFEADKRAVYKHPLFPLLALLFERCEQATQSSDNSTSESFNMDIQAFVQHQERDRKPF
H.magnipapillata    1 ---MENEKVAIYRHPLFPLLAMLLEKCEHATQTSDCPTSDAFDNDIKNFMMQHNREGKPC
N.vectensis         1 ---MENEKVAIYRHPLFPLLAMLLEKCEHATQTSDCPTSDAFDNDIKNFMMQHNREGKPC

Prep2 H.sapiens    61 FSDDPELDNLMVKAIQVLRIHLLELEKVNELCKDFCNRYITCLKTKMHSDNLLRN
Prep2 M.musculus   61 FSDDPELDNLMVKAIQVLRIHLLELEKVNELCKDFCNRYITCLKTKMHSDNLLRN
Prep1 H.sapiens    61 FCEDPETDNLMVKAIQVLRIHLLELEKVNELCKDFCSRYIACLKTKMNSETLLSG
Prep1 M.musculus   61 FCEDPETDNLMVKAIQVLRIHLLELEKVNELCKDFCSRYIACLKTKMNSETLLSG
X.tropicalis       61 FSEDPELDNLMVKAIQVLRIHLLELEKVNELCKDFCNRYITCLKTKMHSDNLLRN
Prep1.1 D.rerio    61 FSEDPDLDNLMVKAIQVLRIHLLELEKVSDLCKDFCSRYISCLKAKMNSETLLSG
Smed-prep          61 FTDNKDLDSLMVKAIQVLRIHLIELRKVNELCRDFCSRYINCLKTKIQSDSMFDD
C.intestinalis     61 IIDNPEIDNLMIKAIQVLRIHLLELEKVNELCKDFCHRYITCLKGKMHSENLLRT
T.castaneum        61 LANEPEIDGLMIKAIQVLRIHLLELEKVQELCKDFCNRYITCLKGKMQSENLLRS
A.mellifera        61 LINDPEIDGLMIKAIQVLRIHLLELEKVQELCKDFCNRYITCLKGKMQSENLLRS
H.magnipapillata   58 FSGDQELDNLIIKAIQVFRIHLLELEKVNELCKDFCQRYIACLKGKMQSENLLR-
N.vectensis        58 FSGDQELDNLIIKAIQVFRIHLLELEKVNELCKDFCQRYIACLKGKMQSENLLR-
```

B

Sequence alignment of the Homeodomain

```
Prep2 H.sapiens     1 KSKNKRGVLPKHATNIMRSWLFQHLMHPYPTEDEKRQIAAQTNLTLLQVNNWFINARRRILQPM
Prep2 M.musculus    1 KSKNKRGVLPKHATNIMRSWLFQHLMHPYPTEDEKRQIAAQTNLTLLQVNNWFINARRRILQPM
Prep1 H.sapiens     1 SSKNKRGVLPKHATNVMRSWLFQHIGHPYPTEDEKKQIAAQTNLTLLQVNNWFINARRRILQPM
Prep1 M.musculus    1 SSKNKRGVLPKHATNVMRSWLFQHIGHPYPTEDEKKQIAAQTNLTLLQVNNWFINARRRILQPM
Prep1.1 D.rerio     1 SPKNKRGVLPKQATNVMRSWLFQHIAHPYPTEEEKKQIATQTNLTLLQVNNWFINARRRILQPM
X.tropicalis        1 KSKNKRGVLPKHATNIMRSWLFQHLMHPYPTEDEKRQIAAQTNLTLLQVNNWFINARRRILQPM
Smed-prep           1 PTKQKRGVLPKKATQIMKQWLFQHLVHPYPTEDEKRQIATQTNLTLLQVNNWFINARRRILQPM
C.intestinalis      1 GRKTKRGVLPKQATEILRSWLFSHIVHPYPTEDEKRSLATQTNLTLLQVNNWFINARRRILQPM
T.castaneum         1 -RKQKRGVLPKHATSVMRSWLFQHLVHPYPTEDEKRHIAAQTNLTLLQVNNWFINARRRILQPM
A.mellifera         1 KGRQKRGVLPKQATSIMRTWLFEHLVHPYPTEDEKRQIASQTNLTLLQVNNWFINARRRILQPM
H.magnipapillata    1 TVKSKRGVLPKQATSIMKTWLFQHIMHPYPTEDEKRSIAQQTNLTILQVNNWFINARRRILQPM
N.vectensis         1 TVKSKRGVLPKQATSIMKTWLFQHIMHPYPTEDEKRSIAQQTNLTILQVNNWFINARRRILQPM
```
