# Supplementary figures and images for: The TALE Class Homeobox Gene Smed-prep Defines the Anterior Compartment for Head Regeneration
Source: PLoS Genet. 2010 Apr 22;6(4):e1000915. doi: 10.1371/journal.pgen.1000915 (PMC2858555; doi:10.1371/journal.pgen.1000915)

Figure S2, related to Figure 2

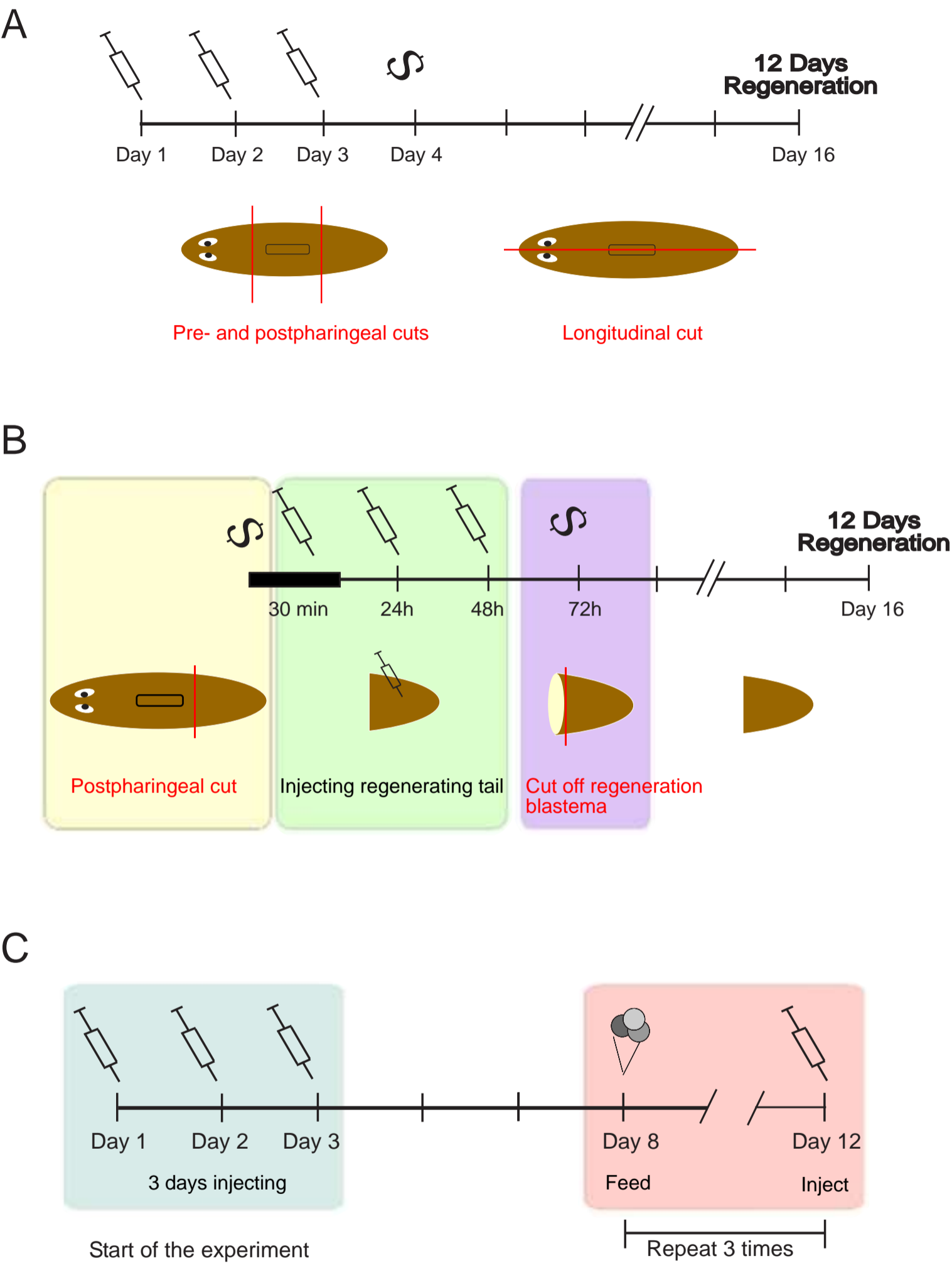

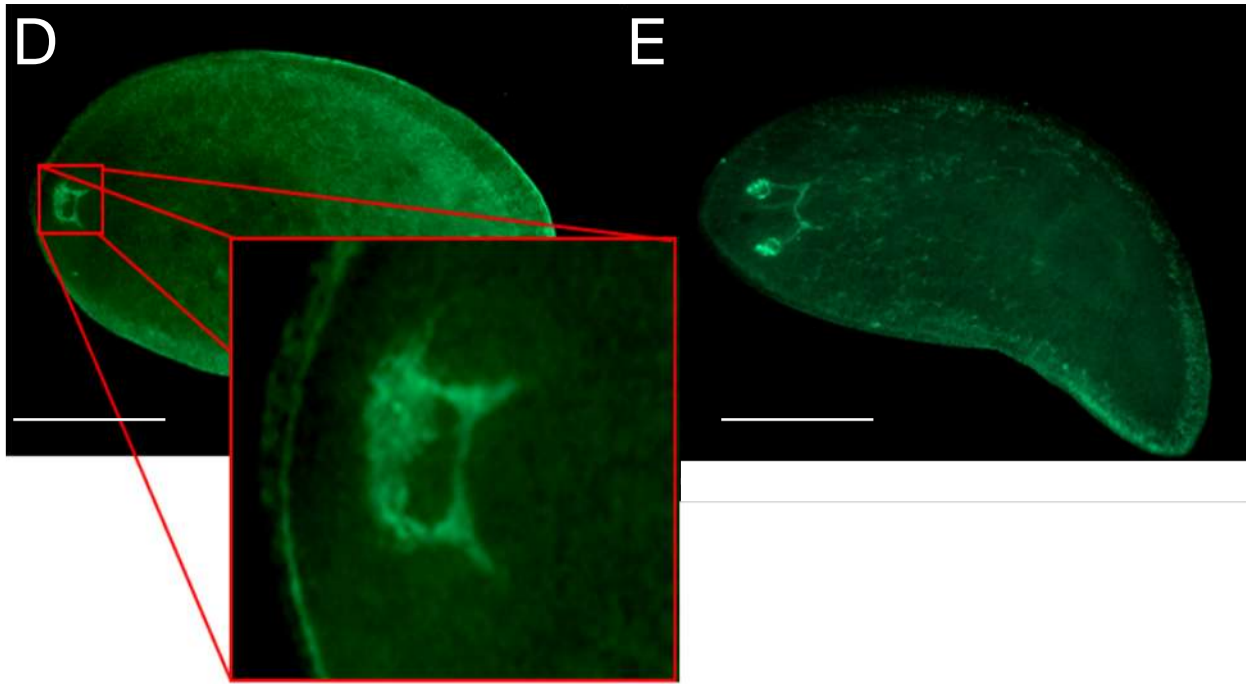

*gfp(RNAi)*

*Smed-prep(RNAi)*

*Smed-slit*

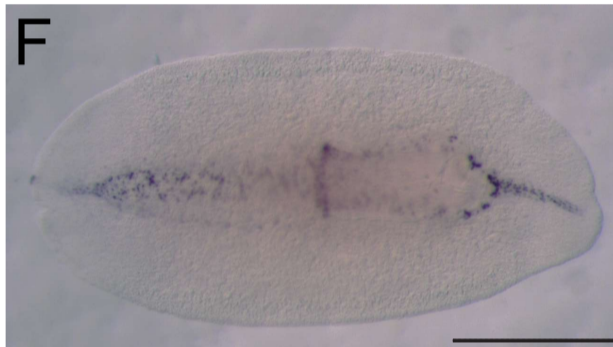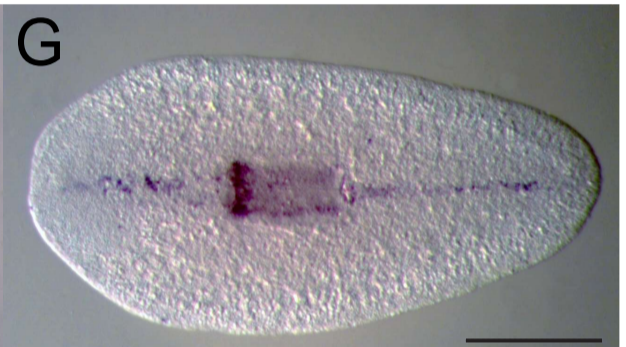

*Smed-sFRP-1*

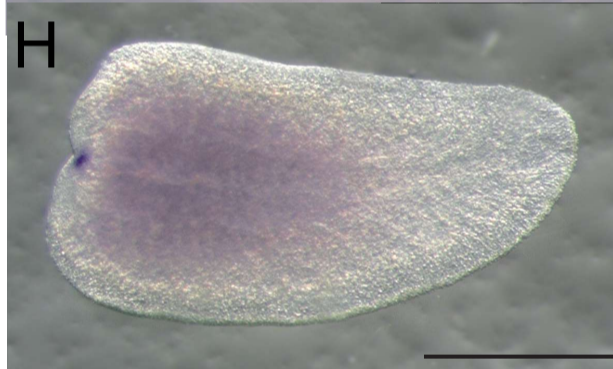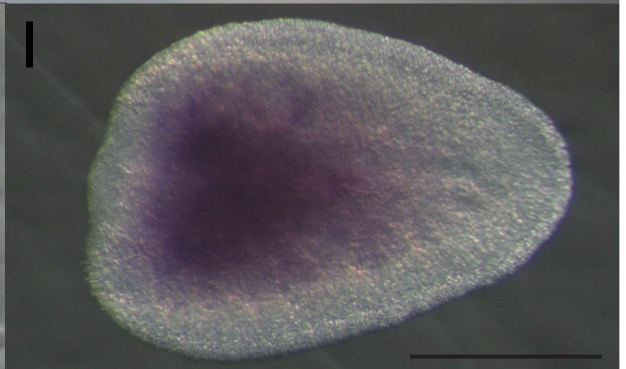

*Smed-HoxD*

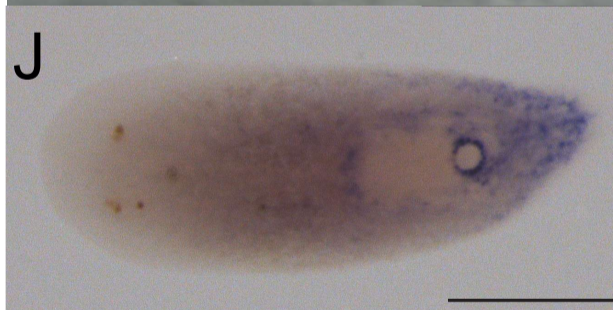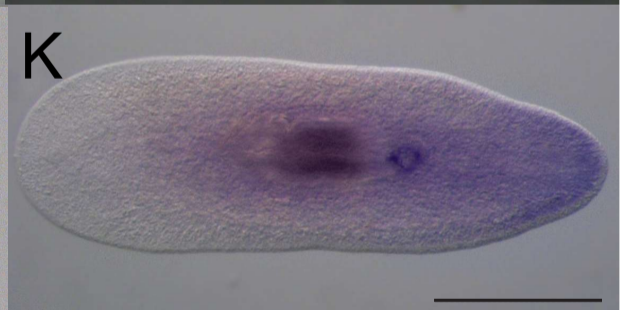

*Smed-Tcen49*

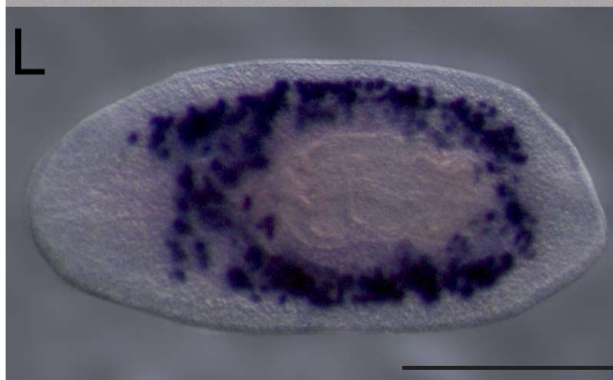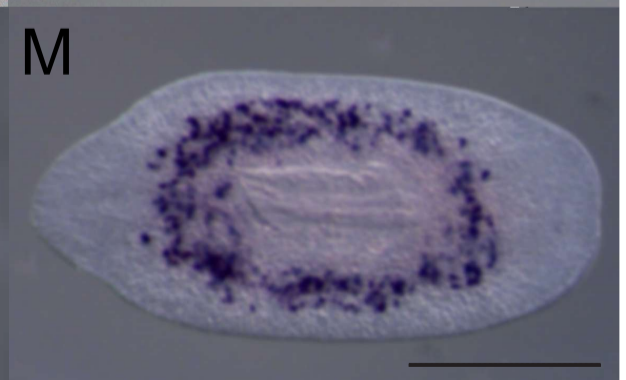

Supplement: Figure S2 — RNAi protocols and characterization of Smed-prep function. Explanation of RNAi injection schemes and further analysis of Smed-prep function. Figurative explanation of RNAi injection and amputation protocols used for assaying Smed-prep function. In the standard protocol animals receive 3×32 nl injections of dsRNA at 1 μg/μl for three consecutive days before pre- and post- pharyngeal or longitudinal amputations are performed (A). To assay the effect of Smed-prep(RNAi) specifically during regeneration animals tails are amputated and injected 3 times with 3×32 nl injections of dsRNA at 1 μg/μl as depicted. The animals are then re-amputated (B). Homeostasis experiments were conducted for 28 days or longer. Initially animals were injected as in (A) but instead of being amputated they were left intact, fed and injected with a single set of 3×32 nl injections of dsRNA at 1 μg/μl for the subsequent weeks. Staining with the anti-arrestin VC-1 monoclonal antibody against the photoreceptor neurons shows that Smed-prep(RNAi) animals have only one photoreceptor, which appears to be a fusion of two normal eyes (D). gfp(RNAi) animals always regenerate a normal visual system (E). The midline of Smed-prep(RNAi) animals (G) seems normal and Smed-slit expression that labels cells in the midline of gfp(RNAi) animals (F) is unaffected. The expression of Smed-sFRP-1 appears early during anterior regeneration. At 24 hours of regeneration it can already be seen in the blastema in gfp(RNAi) animals (H). In Smed-prep(RNAi) animals expression is not detected in tail pieces even when the sample is left to develop until background is very high (I). The expression of HoxD is detected in the tail parenchyma up to the mouth of the pharynx, in the mouth itself and in a few scattered cells just anterior to the pharynx in gfp(RNAi) animals (J). There is no ectopic expression detected in the head of Smed-prep(RNAi) animals (K). The normal expression domain of Smed-Tcen49 in scattered cell clusters in t [file pgen.1000915.s002.pdf]
